# Supplementary material for: A Critical View of the Application of the APEX Software (Aqueous Photochemistry of Environmentally-Occurring Xenobiotics) to Predict Photoreaction Kinetics in Surface Freshwaters
Source: Molecules. 2019 Dec 18;25(1):9. doi: 10.3390/molecules25010009 (PMC7017383; doi:10.3390/molecules25010009)
Supplement: Supplementary file 1 [file molecules-25-00009-s001.zip › 3.2.4_gcc-4.4.0.zip!/3.2.4_gcc-4.4.0/3.2.4_gcc-4.4.0/doc/octave/html/interpreter/Text-Properties.html]

Text Properties - Untitled


Next: Image Properties,
Previous: Line Properties,
Up: Graphics Object Properties


---

##### 15.2.2.5 Text Properties

`string`: The character string contained by the text object. `units`: May be `"normalized"` or `"graph"`. `position`: The coordinates of the text object. `rotation`: The angle of rotation for the displayed text, measured in degrees. `horizontalalignment`: May be `"left"`, `"center"`, or `"right"`. `color`: The color of the text. See Colors. `fontname`: The font used for the text. `fontsize`: The size of the font, in points to use. `fontangle`: Flag whether the font is italic or normal. Valid values are 'normal', 'italic' and 'oblique'. `fontweight`: Flag whether the font is bold, etc. Valid values are 'normal', 'bold', 'demi' or 'light'. `interpreter`: Determines how the text is rendered. Valid values are 'none', 'tex' or 'latex'.

All text objects, including titles, labels, legends, and text, include
the property 'interpreter', this property determines the manner in which
special control sequences in the text are rendered. If the interpreter
is set to 'none', then no rendering occurs. At this point the 'latex'
option is not implemented and so the 'latex' interpreter also does not
interpret the text.

The 'tex' option implements a subset of TeX functionality in the
rendering of the text. This allows the insertion of special characters
such as Greek or mathematical symbols within the text. The special
characters are also inserted with a code starting with the back-slash
(\) character, as in the table tab:extended.

In addition, the formatting of the text can be changed within the string
with the codes

|  |  |  |  |
| --- | --- | --- | --- |
|  | \bf | Bold font |  |
|  | \it | Italic font |  |
|  | \sl | Oblique Font |  |
|  | \rm | Normal font |  |

These are be used in conjunction with the { and } characters to limit
the change in the font to part of the string. For example

```
     xlabel ('{\bf H} = a {\bf V}')
```

where the character 'a' will not appear in a bold font. Note that to
avoid having Octave interpret the backslash characters in the strings,
the strings should be in single quotes.

It is also possible to change the fontname and size within the text

|  |  |  |  |
| --- | --- | --- | --- |
|  | \fontname{fontname} | Specify the font to use |  |
|  | \fontsize{size} | Specify the size of the font to use |  |

Finally, the superscript and subscripting can be controlled with the '^'
and '\_' characters. If the '^' or '\_' is followed by a { character,
then all of the block surrounded by the { } pair is super- or
sub-scripted. Without the { } pair, only the character immediately
following the '^' or '\_' is super- or sub-scripted.

|  |  |  |  |  |
| --- | --- | --- | --- | --- |
|  | \forall | \exists | \ni |  |
|  | \cong | \Delta | \Phi |  |
|  | \Gamma | \vartheta | \Lambda |  |
|  | \Pi | \Theta | \Sigma |  |
|  | \varsigma | \Omega | \Xi |  |
|  | \Psi | \perp | \alpha |  |
|  | \beta | \chi | \delta |  |
|  | \epsilon | \phi | \gamma |  |
|  | \eta | \iota | \varphi |  |
|  | \kappa | \lambda | \mu |  |
|  | \nu | \o | \pi |  |
|  | \theta | \rho | \sigma |  |
|  | \tau | \upsilon | \varpi |  |
|  | \omega | \xi | \psi |  |
|  | \zeta | \sim | \Upsilon |  |
|  | \prime | \leq | \infty |  |
|  | \clubsuit | \diamondsuit | \heartsuit |  |
|  | \spadesuit | \leftrightarrow | \leftarrow |  |
|  | \uparrow | \rightarrow | \downarrow |  |
|  | \circ | \pm | \geq |  |
|  | \times | \propto | \partial |  |
|  | \bullet | \div | \neq |  |
|  | \equiv | \approx | \ldots |  |
|  | \mid | \aleph | \Im |  |
|  | \Re | \wp | \otimes |  |
|  | \oplus | \oslash | \cap |  |
|  | \cup | \supset | \supseteq |  |
|  | \subset | \subseteq | \in |  |
|  | \notin | \angle | \bigrightriangledown |  |
|  | \langle | \rangle | \nabla |  |
|  | \prod | \surd | \cdot |  |
|  | \neg | \wedge | \vee |  |
|  | \Leftrightarrow | \Leftarrow | \Uparrow |  |
|  | \Rightarrow | \Downarrow | \diamond |  |
|  | \copyright | \lfloor | \lceil |  |
|  | \rfloor | \rceil | \int |  |

**Table 15.1: Available special characters in TeX mode**

A complete example showing the capabilities of the extended text is

```
     x = 0:0.01:3;
     plot(x,erf(x));
     hold on;
     plot(x,x,"r");
     axis([0, 3, 0, 1]);
     text(0.65, 0.6175, strcat('\leftarrow x = {2/\surd\pi',
     ' {\fontsize{16}\int_{\fontsize{8}0}^{\fontsize{8}x}}',
     ' e^{-t^2} dt} = 0.6175'))
```
